# Integrated multi-omics reveals the activated retinal microglia with intracellular metabolic reprogramming contributes to inflammation in STZ-induced early diabetic retinopathy

**DOI:** 10.3389/fimmu.2022.942768

**Published:** 2022-09-02

**Authors:** Kangjia Lv, Hui Ying, Guangyi Hu, Jing Hu, Qizhi Jian, Fang Zhang

**Affiliations:** ^1^ National Clinical Research Center for Eye Diseases, Shanghai General Hospital, Shanghai Jiao Tong University School of Medicine, Shanghai, China; ^2^ Shanghai Key Laboratory of Fundus Diseases, Shanghai General Hospital, Shanghai Jiao Tong University School of Medicine, Shanghai, China; ^3^ Shanghai Engineering Center for Visual Science and Photomedicine, Shanghai General Hospital, Shanghai Jiao Tong University School of Medicine, Shanghai, China; ^4^ Shanghai Engineering Center for Precise Diagnosis and Treatment of Eye Diseases, Shanghai General Hospital, Shanghai Jiao Tong University School of Medicine, Shanghai, China

**Keywords:** Diabetic retinopathy, inflammation, multi-omics, microglia, metabolic reprogramming

## Abstract

Diabetic retinopathy (DR) is the leading cause of visual impairment and blindness among working-age people. Inflammation is recognized as a critical driver of the DR process. However, the main retina-specific cell type producing pro-inflammatory cytokines and its mechanism underlying DR are still unclear. Here, we used single-cell sequencing to identify microglia with metabolic pathway alterations that were the main source of IL-1β in STZ-induced DR mice. To profile the full extent of local metabolic shifts in activated microglia and to reveal the metabolic microenvironment contributing to immune mechanisms, we performed integrated metabolomics, lipidomics, and RNA profiling analyses in microglia cell line samples representative of the DR microenvironment. The results showed that activated microglia with IL-1β increase exhibited a metabolic bias favoring glycolysis, purine metabolism, and triacylglycerol synthesis, but less Tricarboxylic acid (TCA). In addition, some of these especially glycolysis was necessary to facilitate their pro-inflammation. These findings suggest that activated microglia with intracellular metabolic reprogramming in retina may contribute to pro-inflammation in the early DR.

## Introduction

Diabetic retinopathy (DR) is the most common reason of vision loss among diabetes and the leading cause of vision impairment and blindness among adults aged 20–65 years (1–3). With the rising prevalence of diabetes expected to reach 642 million by 2040, the International Diabetes Federation estimates that 35% (224 million) of them will progress to some form of DR and that 11% (70 million) will have sight-threatening DR (2, 4). The current studies mainly focus on proliferative DR with vascular pathogenesis at the advanced stage of DR, and multiple clinical trials have demonstrated the efficacy and safety of intraocular anti-VEGF therapy in nearly half of patients with advanced DR (5, 6). Fewer treatments are available for the early stages of DR to prevent alterations of the microvascular damage and thereby preserve visual function for the mechanisms unclear (7, 8).

The correlation between DR and inflammation had been discovered when patients with diabetes treated with salicylates for rheumatoid arthritis had fewer complications than untreated patients (9). Early retinal inflammatory processes are now well established to play a central role in the pathogenesis of DR (10). To explore the underlying mechanisms, previous studies have indicated that diabetes mellitus–associated tissue hypoxia and immune adaptations could induce expression of numerous inflammatory cytokines and chemokines responsible for DR development (11). Various pro-inflammation cytokines including Interleukin-1β, Tumor Necrosis Factor alpha, and Interleukin-6 were expressed in the whole retina of patients with DR (12–15). IL-1β and TNF-α have been reported to promote angiogenesis and ocular neovascularization effectively (16, 17), and IL-6 regulates immune cell function, increases vascular permeability, and promotes retinal neovascularization (18). Moreover, tetracycline derivative minocycline could reduce the level of retinal inflammatory factors in the retina of DR rats and is on clinical trials phase II now (19, 20). The anti-inflammatory salicylic acid has also been reported to inhibit early damage in animals with DR (21). Moreover, the IL-1β receptor antagonist anakinra could alleviates streptozotocin (STZ)–induced endothelial dysfunction in diabetic rats (22). Therefore, to further identify the cellular targets in the retina that are the main source of pro-inflammation cytokines and to reveal its underlying mechanisms would decipher the precise mechanisms of inflammation and provide potential approaches for early DR treatment.

In the early stage of DR, glial cell activation, leukocyte stasis adhesion, and blood-retinal barrier disruption may play a synergistic role in retinal inflammation (10, 23). Retinal glial cells, including macroglia (Müller cells and astrocytes) and microglia, provide structural and metabolic support for retinal function by interacting with the cells that transudative the photons (24–26). Although histopathological studies show that early structural changes of macroglia and microglia occur simultaneously in diabetic rats (27), human DR studies indicated that rapid activation of microglia and their typical morphological changes could be detected before macroglia activation (28). Moreover, the increased expression of IL-1β appeared in endothelial cells by comparing retinal microglia, vascular endothelial cells, astrocytes, and Müller cells with high-glucose stimuli *in vitro* (29). However, the main type of cells producing the retinal pro-inflammatory factors *in vivo* at the early stage of DR remains unclear.

Single-cell RNA sequencing (scRNA-Seq) is powerful to identify specific cell types and to reveal molecular mechanisms underlying biological and pathological processes by profiling gene expression at single-cell resolution (30). Several scRNA-Seq studies have focused on deepening the understanding of cells in the physiology and pathology of retina. A single-cell transcriptome atlas of the adult human retina was profiled by scRNA-Seq to fundamentally understand retinal biology (25). To track the retina development, scRNA-Seq has been utilized to uncover distinct the subsets of early and late-stage retinal progenitors and evolutionarily conserved and species-specific cell type and to identify mechanisms involved through stark differences in gene expression patterns between subclusters (31). scRNA-Seq maps cell-type–specific expression of Rgcc gene in capillary endothelial cells associated with human retinal diseases (32). Currently, scRNA-Seq provides a platform to decipher the retinal inflammatory cells as macroglia, microglia, and monocytes at the advanced stage of DR in the Akimba mice model (33). Our previous study utilized scRNA-Seq to overview the pathological alterations of retinal cells in STZ-induced diabetic retinas (24). We found 11 cell types including six classes of retinal neurons (rods, cones, bipolar cells, amacrine cells, horizontal cells, and retinal ganglion cells), three types of glia (microglia, Müller cells, and astrocytes), and two classes of blood vascular structures (endothelial cells and pericytes). These retinal cells were further divided into more subtypes, and we observed and defined a subpopulation of endothelial cells (DRECs) with IL-17 signaling pathways significant activation. It is documented that glial cell activation, leukocyte stasis adhesion, and blood-retinal barrier disruption may play a synergistic role in retinal inflammation in the early stage of DR (23, 24). However, the precise cell types and mechanisms governing the inflammation in the early stage of DR are incompletely understood.

In this study, we utilized our scRNA-Seq analysis to present the expression of pro-inflammation cytokines including IL-1β, TNF, and IL-6 that were reported to express in the whole retina of patients with DR and identified a subcluster of activated retinal microglia (ARM) with immunometabolic features as the main source of IL-1β and TNF at the early stage of DR in STZ-induced DR mice. Integrated metabolomics and lipidomics profiled the intracellular metabolic microenvironment of activated microglia and showed that activated microglia with IL-1β increase exhibited a metabolic bias favoring glycolysis, purine metabolism, and TAG synthesis but less TCA. Moreover, some metabolic reprogramming particularly glycolysis is necessary to facilitate their pro-inflammation. We thus propose that the activated microglia with intracellular metabolic reprogramming in retina contribute to pro-inflammation in DR.

## Materials and methods

### Animals

All animal experiments were approved by the Animal Care and Use Committee of Shanghai General Hospital. DR mice were induced by intraperitoneal (IP) injection of STZ (Sigma, USA, cat. no. V900890). In detail, male C57BL/6J mice (8–10 weeks of age) were overnight fasting before treatment with freshly prepared STZ in sodium citric buffer (pH 4.2) and administrated to mice through IP injection at 55 mg/kg body weight for consecutive 5 days, and the citrate buffer was injected for the control group. Body weight and blood glucose levels were monitored weekly after the STZ treatment using electronic balance (Sartorius) and the Accu-Chek active glucometer (Roche). Only animals with persistently elevated blood glucose of more than 300 mg/dl were considered diabetic mice and used for the study. Following a 16-week period of sustained high blood glucose concentration, electroretinograms (ERGs), and retinal samples for RT-PCR and immunofluorescence were measured and collected, respectively. As described in our previous study, retinal samples for scRNA-Seq were collected from mice that sustained a high blood glucose concentration for 25 weeks (24).

### Electroretinogram

Before the ERG recordings, mice were dark-adapted overnight. Mice were anesthetized by IP injection of 1.5% sodium pentobarbital. The mouse with the dilated pupil were placed on the table, and then the corneal contact electrode, reference electrode, and ground electrode were placed on the mouse. A Phoenix Ganzfeld System (Cold Spring Harbor Corp, USA) was used to record the scotopic a/b-wave at a stimulus intensity of 4 log cd.s/m^2^.

### Single-cell sequencing and data analysis

Single-cell transcriptome files of murine retinas were from our previous research paper (24). In brief, the retinas from STZ-induced mice and control were digested with 500 μl of 2 U of papain (Worthington) solution containing 0.5 mM EDTA and 2.5 mM L-Cysteine at 37°C for 10 min. Then, 750 μl of AMES (Sigma, USA, cat. no. A1420) medium containing 10% Fetal bovine serum (FBS) was added to blunt the reaction, followed by digestion with 7.5 U of DNase I (Roche, Switzerland, cat. no. 4536282001) at 37°C for 5 min. The tissues were gently pipetted up and down several times to generate single-cell suspension. To reduce the number of rods, the single-cell suspension was centrifuged at 300*g* for 5 min and then re-suspended in 200 μl of AMES including 0.2% Bovine serum albumin (BSA). Then, the cell suspension was sequentially incubated with CD73 antibody (BD Biosciences, cat. no. 561014, USA) and anti-rat Immunoglobulin G (IgG) microbeads (Miltenyi Biotec, Germany, cat. no. 130-048-501). After the cells were washed once with 0.2% FBS-containing AMES, the CD73^+^ cells were removed by MACS separator (Miltenyi Biotec, Germany, cat. no. 130-092-168). The cell number and cell viability were determined by Calcein Calcein AM and Propidium iodide (AM-PI) staining.

Data were filtered by the R package Seurat (v4.0.4) with the following settings: a gene count per cell >200 and <6,500, a percentage of mitochondrial genes <10%, and the unique molecular identifiers (UMIs) count >1,000 and <20,000 per cell. After filtering, we used 14,355 retina cells for further clustering and analysis with the Seurat package (34). Top 30 dimensions were used to generate final clusters using principal component analysis (PCA) and graph-based clustering. The t–Distributed Stochastic Neighbor Embedding (t-SNE) was used to reduced dimensionality, and a total of 40 clusters were identified and finally defined by canonical cell markers. The Seurat–Bimod statistical test was used to detect differentially expressed genes (DEGs) in microglia cells between the control and STZ groups. The genes with an absolute log_2_ fold change greater than 1.5 were identified as DEGs between the two cell groups. The adjusted p-value for multiple testing was calculated using the Benjamini–Hochberg correction. The Kyoto Encyclopedia of Genes and Genomes (KEGG) pathway enrichment analysis was done using the hypergeometric test in R. Significantly enriched KEGG pathways were selected by a threshold False discovery rate (FDR) (adjusted p-value) < 0.05.

### Immunofluorescence of retina

The mice were anesthetized with the IP injection of 1.5% sodium pentobarbital. Eyes were dissected out and post-fixed in 4% Paraformaldehyde solution (PFA) at 4°C for 1 h. For frozen sections, tissues were immersed in 10% and 20% sucrose for 30 min and in 30% sucrose overnight before sectioning in a cryostat with section thickness of 10 μm for retina. Before staining, retinal sections were incubated with 5% BSA and 1% Triton X-100 in Phosphate-buffered saline (PBS) for 1 h at room temperature, rinsed with the blocking solution, put into the blocking solution containing the primary Ionized calcium-binding adapter molecule 1 (IBA-1) antibody (1:500; Wako, cat. no. 019-19741, Japan), and incubated in a humidified box at 4°C for 12 to 16 h. After several washes, sections were incubated with the Alexa Fluor 488 secondary antibody (1:500, Thermo Fisher Scientific, USA, cat. no. A-11034). In addition, nuclei were stained with Hoechst 33342 (Sangon Biotech, USA, cat. no. E607302). The immunofluorescent images were obtained using a confocal microscope (Leica, Germany).

### Cell culture

BV2 murine microglia were obtained from the Shanghai General Hospital Cell Culture Facility. Cells were grown in modified medium supplemented with 10% fetal bovine serum (BI, USA, cat. no. 04-001-1A) and 1% penicillin/streptomycin and Dulbecco’s modified Eagle’s medium high glucose (Corning, USA, cat. no. 10-013-CV), cultured at 37°C, 5% CO_2_. The cell line was passaged at 70% confluence every 3 days and seeded on 10-cm plates at a density of 1.1 × 10^6^ cells. All the cell studies were performed using BV2 cells between passage 2 and passage 8. BV2 cells were seed six-well plate at a density 1.5 × 10^5^ cells and culture under 21% O_2_ for 24 h prior to hypoxia treatment. The hypoxia chamber (Hariolab, China) inside a CO_2_ incubator was used to perform hypoxia experiments and simulate microglial activation *in vitro*. BV2 subjected to 2-DG (1, 5, and 10 mM; Beyotime, China, cat. no. ST1024) and TAG synthesis inhibitor A922500 (10, 25, 50, and 100 nM; MCE, China, cat. no. S2674) was exposed to 1% O_2_ with 5% CO_2_ for hypoxia for a total exposure time of 24 h. Control BV2 cells were maintained in 21% O_2_ for identical 24 h. To minimize the effect of oxygen present, we performed protein and mRNA extraction as soon as possible after hypoxia treatment.

### RNA sequencing

Total RNA was extracted, and a complementary DNA library was generated using the VAHTS Universal V6 RNA-seq Library Prep Kit for Illumina Kit (Vazyme, China, cat. no. NR604-02) according to the manufacturer’s protocol. The complementary DNA libraries were sequenced using Illumina NovaSeq6000 with 2 × 150 running circles. The raw fastq reads were aligned to mm10 mouse reference genome using STAR aligner (35). FeatureCounts was used to quantify the gene expression levels (36). Only genes with reads count were used for further analysis of differential expression using Deseq2 (37). Genes with expression changes of two-fold compared with control samples were considered to be DEGs. The Gene Ontology and KEGG were analyzed using TopGO for gene enrichment.

### Metabolomics

Cells were added with 500 μl of −80°C pre-chilled 80% mass spectrometry (MS) grade methanol after quick-freezing in liquid nitrogen and then scraped off cells with a cell scraper and transferred to a 1.5-ml Eppendorf tube (EP) tube. The dishes were then rinsed with another 500 μl of 80% MS grade methanol, transferred to the same EP tubes, and quick-frozen in liquid nitrogen. The collected samples were thawed at 37°C, vortexed for 30s, snap-frozen in liquid nitrogen, thawed at 37°C, repeated five to 10 times, and centrifuged at 4°C and 15,000 rpm for 10 min. Next, 800 μl of the supernatant (depending on the volume added above, avoid using any pellets) was taken and centrifuged for another 10 min at 4°C; if floating lipid-like fraction or cell debris was found (sometimes in the top layer), then they need to be centrifuged for an additional 30 min at a maximum speed at 4°C (if they are difficult to settle, avoid using them when transferring to a new EP tube). Another 750 μl of the supernatant was transferred to a new EP tube, and the sample was dried completely using a speed vac dryer. The dried samples were resuspended in 50–100 μl of 60% acetonitrile/H_2_O (MS grade), pipetted vigorously for 30 s, placed on ice for 30 s, repeated at least five times, and then placed on ice for at least 30 min. The sample was centrifuged at 15,000*g* at 4°C for 10 min; the supernatant was transferred to a new EP tube and centrifuged for 10 min; and then, 40–80 μl of the sample was taken into an MS sample vial.

The target metabolites were analyzed by liquid chromatography–mass spectrometry/mass spectrometry (LC/MS/MS). The separation was performed on 150 × 2.1 mm, 5-µm SeQuant ZIC-pHILIC HPLC Column (Merk, USA). Mobile phase solvents were composed of A = 5 mM ammonium acetate in water and B = 100% acetonitrile. The gradient program was as follows: 0–1 min, 20% B; 1–3 min, 20%–60% B; 3–13 min, 60%–98% B; 13–13.1 min, 98%–20% B; and 13.1–16 min, 20% B. Column temperature was 45°C, and autosampler was charged to 4°C. The flow rate was 0.15 ml/min, and the injection volume was 2 μl. The mass spectrometer was QTRAP 6500 LC-MS/MS System (SCIEX, USA). Sample analysis was performed in positive/negative switching mode. The detection conditions of MRM-MS/MS are defined as follows: curtain gas (CUR), 45 psi; ion spray voltages (IS), 5,500 V (positive) and −4,500 V (negative); temperature (TEM), 350°C; ion source gas 1 (GS1), 45 psi; and ion source gas 2 (GS2), 40 psi. Data were normalized and analyzed by MetaboAnalyst.

### Lipidomics

Hypoxic-treated BV2 cells were washed once with 37°C pre-warmed PBS; 225 μl of −80°C pre-cooled 100% MS grade methanol was added; cells were scraped with a cell scraper and transfered to a 1.5-ml EP tube. The dishes were then rinsed with another 225 μl of 100% MS grade methanol, transferred to the same EP tubes, and quick-frozen in liquid nitrogen.

Methanol of 225 μl was added to the sample EP tube, and then 20 μl (adjusted according to the number of samples) was pipetted from each sample into the QC tube at the maximum vortex speed for 30 s. After adding internal standard (optional), 750 μl of MTBE (methyl tert-butyl ether) was added, vortexed at maximum speed for 30s, and rotated at room temperature for 30 min. Then, 188 μl of mass spectrometry water was added, vortexed for 30s, left at room temperature for 10 min, then centrifuged at low temperature at 15,000*g* at 4°C for 15 min at high speed; 700 μl of supernatant (the sample is divided into lipid phase, water phase, and solid residue from top to bottom) was taken and transferred to a new in the EP tube. The supernatant was dried with a nitrogen blower. Complex solution of 100 μl was adeed (2:1:1 volume ratio of isopropanol:acetonitrile:water), vortexed for 10 s, and centrifuged at 14,000g (4°C) for 10 min to recover lipids. The supernatant was pipetted into a sample tube for machine testing. Separation was achieved on ACQUITY UPLC BEH C18 Column HPLC column (150 × 2.1 mm, 5 μm). The mobile phases employed were 5 mM ammonium acetate in methanol:acetonitrile:water with raio of 1:1:1 (A) and isopropanol with 5 mM ammonium acetate (B). The gradient program and mass spectrometry parameters were similar to metabolomics.

### Quantitative real-time PCR

Total RNA was extracted from mice retina or BV2 cells using the RNA pure Micro Kit following the manufacturer’s protocol (JIANSHI Biotech, China, cat. no. TR50). The quality and concentration of RNA samples were assessed with the NanoDrop 2000c spectrophotometer (Thermo Fisher Scientific, USA). cDNA synthesis using RNA samples with a ratio of 260/280 > 2.0 and OD 260/230 ratio close to 2.0. One microgram of RNA was used for reverse transcription using the RT Master Mix (Vazyme, China, cat. no. R212). Quantitative real-time PCR was performed as triplicates in 96-well plates in a 10-μl reaction volume containing 5 μl of 2 × SYBR Green qPCR SuperMix (Vazyme, China, cat. no. Q711) and 0.2 µM primers using the ViiA™ 7 Real-Time PCR System (Applied Biosystems, USA). The PCR protocol used was 40 cycles of 35 s at 95°C, 5 s at 60°C, and 34 s at 72°C. Primers used were follows:

β-actin (Actin): (forward) 5′-CATTGCTGACAGGATGCAGAAGG-3′ and (reverse) 5′- TGCTGGAAGGTGGACAGTGAGG-3′; IL-1β: (forward) 5′-TGGACCTTCCAGGATGAGGACA-3′ and (reverse) 5′-GTTCATCTCGGAGCCTGTAGTG-3′; Tnf: (forward) 5′- CATCTTCTCAAAATTCGAGTGACAA-3′ and (reverse) 5′-TGGGAGTAGACAAGGTACAACCC-3′; IL6: (forward) 5′-TACCACTTCACAAGTCGGAGGC-3′ and (reverse) 5′-CTGCAAGTGCATCATCGTTGTTC-3′. Relative gene mRNA expressions were determined using the 2^−ΔΔCt^ method, with normalization to Actin.

### Western blot

BV2 cells were lysed in RIPA buffer and determined with Bradford reagent (Beyotime, cat. no. P0006C). The protein samples were separated on 10% tris-glycine polyacrylamide gels (Sangon Biotech, China, cat. no. C681102). Transfer proteins to nitrocellulose membranes with the Transfer-Blot Turbo Transfer System (Bio-Rad, USA). Membranes were blocked with Protein-Free Rapid Blocking Buffer (Epizyme Biotech, China, cat. no. PS108P) for 15 min and incubated overnight with primary antibodies. The following primary antibodies were used: anti-tubulin antibody (1:1,000; ABclonal, China, cat. no. AC026) and anti-IL-1β (1:1,000; RD, USA, cat. no. AF-401). Tubulin was detected using goat anti-rabbit secondary antibody (1:5,000; Jackson Immuno Research Labs, USA, cat. no. 111-035-003), and IL-1β was detected using donkey anti-goat secondary antibody (1:2,000; Beyotime, China, cat. no. A0181). Signals were visualized with the ChemiDoc Imaging System (Bio-rad).

### Statistical analysis

Results are presented as the mean ± standard error of the mean (SEM). Difference between groups was assessed by ANOVA or Student’s t-test. GraphPad Prism 9 was used for all statistical analyses.

## Results

### Pro-inflammatory cytokine IL-1β increases in STZ-induced diabetic retina

**Figure 1:**
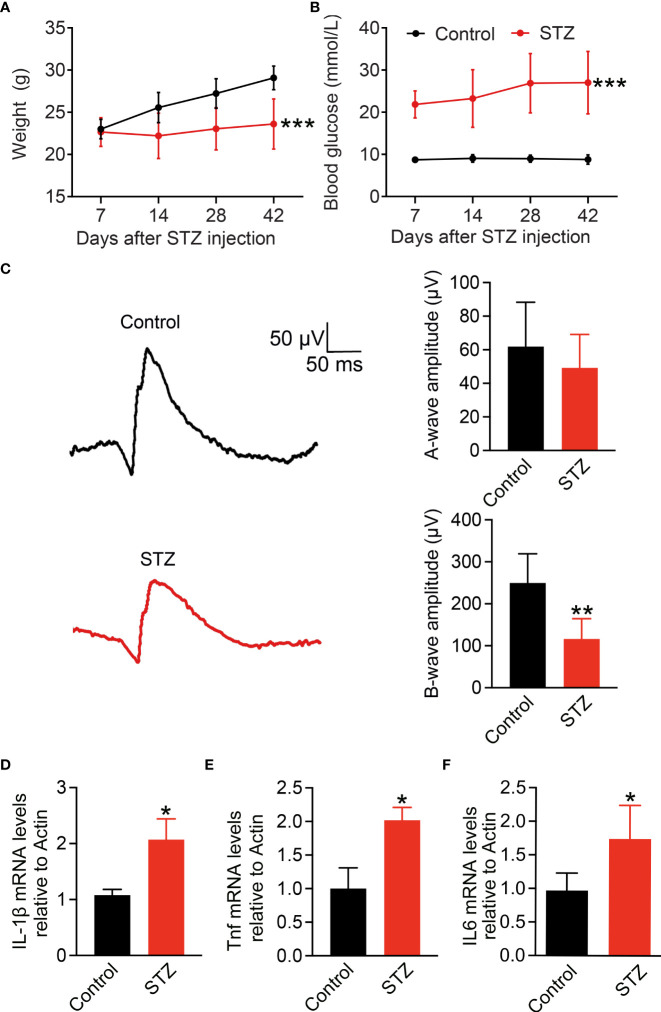
Pro-inflammatory cytokine IL-1β increases in STZ-induced diabetic retina. **(A,B)** Body weight and blood glucose levels were measured weekly after STZ or PBS injection in C57BL/6 male mice (n = 24). **(C)** Detection of vision functional changes in diabetic retina using representative scotopic ERG. Traces recorded at 4 log cd.s/m^2^ flash showing mean responses from non-diabetic control (black) and diabetic mice (red). Histograms of a and b waves of all animals studied. **(D–F)** Retinal IL-1β, Tnf, and IL6 expressions at mRNA level were detected using RT-PCR (n = 4); *p < 0.05, **p < 0.01 and ***p < 0.001.

To address the main source of pro-inflammatory cytokines in the retina of early DR, here, we established the early DR mice model with an inflammatory response first. Diabetes was induced in mice by five consecutive daily low-dose STZ injections. Body weight and blood glucose levels of mice were measured every 7 days (**Figures 1A, B**). Fourteen days after STZ injection, mice with body weight significantly decreased and blood glucose raised to 300 mg/dl were considered to have diabetes. The full-field ERG can reveal early functional changes in the retinas of human and mice diabetics (38, 39). To confirm the visual function affected in the STZ-induced mice model, latencies of the oscillatory potentials were measured by ERG, and the amplitudes of the b-wave were reduced after 4 months of STZ treatment (**Figure 1C**). Chronic inflammation is the feature of diabetes-associated retinal perturbations (11, 40). Retinal pro-inflammatory factors including IL-1β, Tnf, and IL6 were measured by RT-PCR (**Figures 1D–F**). Compared with the control mice, diabetic mice showed significantly enhanced expression of IL-1β, Tnf, and IL6 after 4 months of STZ treatment. These data indicate the early DR mice model with inflammatory response characteristics is successfully established.

### scRNA-Seq identifies activated retinal microglia as the main source of IL-1β in STZ-induced DR mice retina

**Figure 2:**
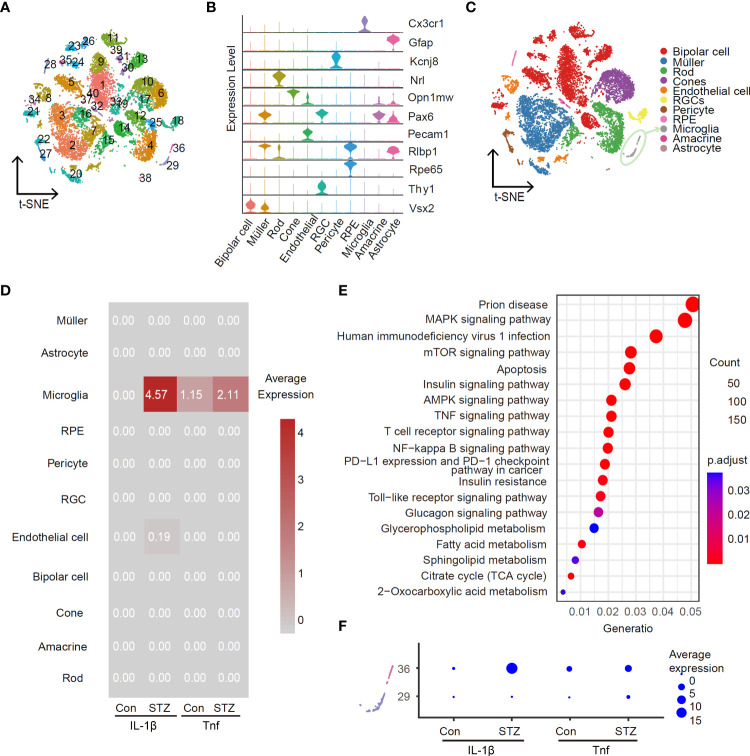
Activated retinal microglia is the main source of IL-1β in the retina of STZ-induced DR mice. Feature plot in the t-SNE map of retina cells from healthy and STZ-induced diabetic mice revealing forty clusters **(A)** and eleven cell types **(C)** by scRNA-Seq program (after quality control, there were 6,126 cells in the control group and 7,851 in the STZ group). RGC, retinal ganglion cell; RPE, retinal pigment endothelial cell. **(B)** Violin plot of top marker genes for each cell subgroup. **(D)** Average expression of IL-1β and Tnf from the control and STZ groups in all subpopulations as visualized by heatmap. **(E)** KEGG enrichment analysis of differentially expressed genes in microglia cells between the control and STZ groups. **(F)** Average expression of IL-1β and Tnf in microglia subclusters 29 and 36 from the control and STZ groups as visualized by dotplot.

To identify the main retina-specific cell types producing pro-inflammation cytokines at the early stage of DR, we utilized scRNA-Seq to profile gene expression at retinal single-cell resolution for STZ-induced mice and control group, respectively. To enhance the ratio of retinal immune cells, we used CD73 antibody to remove rods that account for over 70% of all murine retinal cells from cell suspension as our previous study (24). In total, we captured a total of 6,126 cells from control and 7,851 cells from the STZ retina. After re-filtering, 40 clusters were identified in total (**Figure 2A**). Representative marker genes of cell types revealed these 40 clusters into 11 cell types in the retina (**Figures 2B, C**). Interestingly, IL-1β and Tnf were highly enriched in microglia, when testing the average expression level of pro-inflammatory cytokines (**Figure 2D**). In addition, the expression of IL-1β and Tnf was significantly higher in the STZ group microglia than in control. We subsequently performed KEGG analysis using DEGs in microglia cells between control and STZ groups. We found homologous results for the amount of inflammatory-associated signaling pathways like mammalian target of rapamycin (mTOR), mitogen-activated protein kinase (MAPK), and nuclear factor kappa B (NF-kB) pathways shown in **Figure 2E**. To further identify the subcluster of inflammatory microglia, we investigated subclusters of microglia in **Figure 2A** named clusters 29 and 36 and found cluster 36 with the majority of IL-1β and Tnf expression (**Figure 2F**). These results indicated that cluster 36 as the ARM is the major source of retinal inflammatory cytokines at the early stage of DR.

### Activated retinal microglia in the retina of STZ-induced DR mice show metabolic and inflammatory disturbances

**Figure 3:**
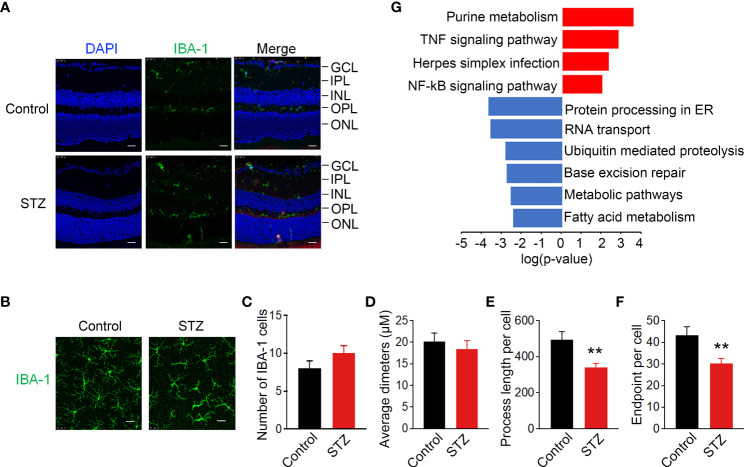
Activated retinal microglia in STZ-induced DR mice with metabolic and inflammatory disturbances. **(A)** Representative retinal cross sections of STZ stained with 6-Diamidino-2-Phenylindole (DAPI) (blue) and IBA-1 (green) showed activated microglia translocation to the ONL. Scale bar indicates 100 μm. ONL, outer region of outer nuclear layer; OPL, outer plexiform layer; INL, inner region of outer nuclear layer; IPL, inner plexiform layer; GCL, ganglion cell layer. **(B)** Morphological changes in microglia immunolabelled for IBA-1 (green) in retinas of diabetic mice indicated activated microglia with shortened processes. Scale bar indicates 25 μm. **(C–F)** Summary data of the number of microglia per field, average diameters of microglia, process length, and number of endpoints per cell. n = 3, **p < 0.01. **(G)** KEGG analysis identified metabolic and inflammatory-related pathways enriched in activated retinal microglia (ARM) according to the differentially expressed genes in cluster 36 from control and STZ retina. **p < 0.01.

As mentioned in the literature, activated microglia in the retina undergo redistribution from the inner to outer layers of the retina in diabetic mice (41). To verify the existence of ARM in the retina of STZ-induced mice, immunostaining of IBA-1 as a specific marker of microglia was used to detect the location and morphology of microglia. In the retina cryosections of non-diabetic mice, the vast majority of microglia was found in the inner plexiform layer (IPL) and outer plexiform (OPL) and was absent from the outer nuclear layer (ONL). Whereas in diabetic mice after 4 months of STZ treatment, the activated retina microglia could be observed traversing the outer retina in the ONL (**Figure 3A**). To further quantify the activation of microglia, we performed IBA-1 immunostaining in retinal flat mounts. IBA-1–positive microglia displayed the ramified morphology typical of resting microglia in control, whereas microglia in the diabetic retina were larger and displayed a more irregular, amoeboid morphology with decreased process length per cell and number of endpoints per cell (**Figures 3B–F**). To further explore the possible regulatory mechanisms within ARM, KEGG analysis was utilized and suggested that metabolic and inflammation-related pathways were enriched in inflammatory microglia cluster 36 as shown in **Figure 2C** (**Figure 3G**). These results signified that ARM with a ramified morphology and migration toward the ONL in the STZ-induced mice model of DR may have metabolic and inflammatory disturbances.

### Hypoxia-induced BV2 microglia activation with metabolic and inflammatory alterations to mimic activated retinal microglia in DR

**Figure 4:**
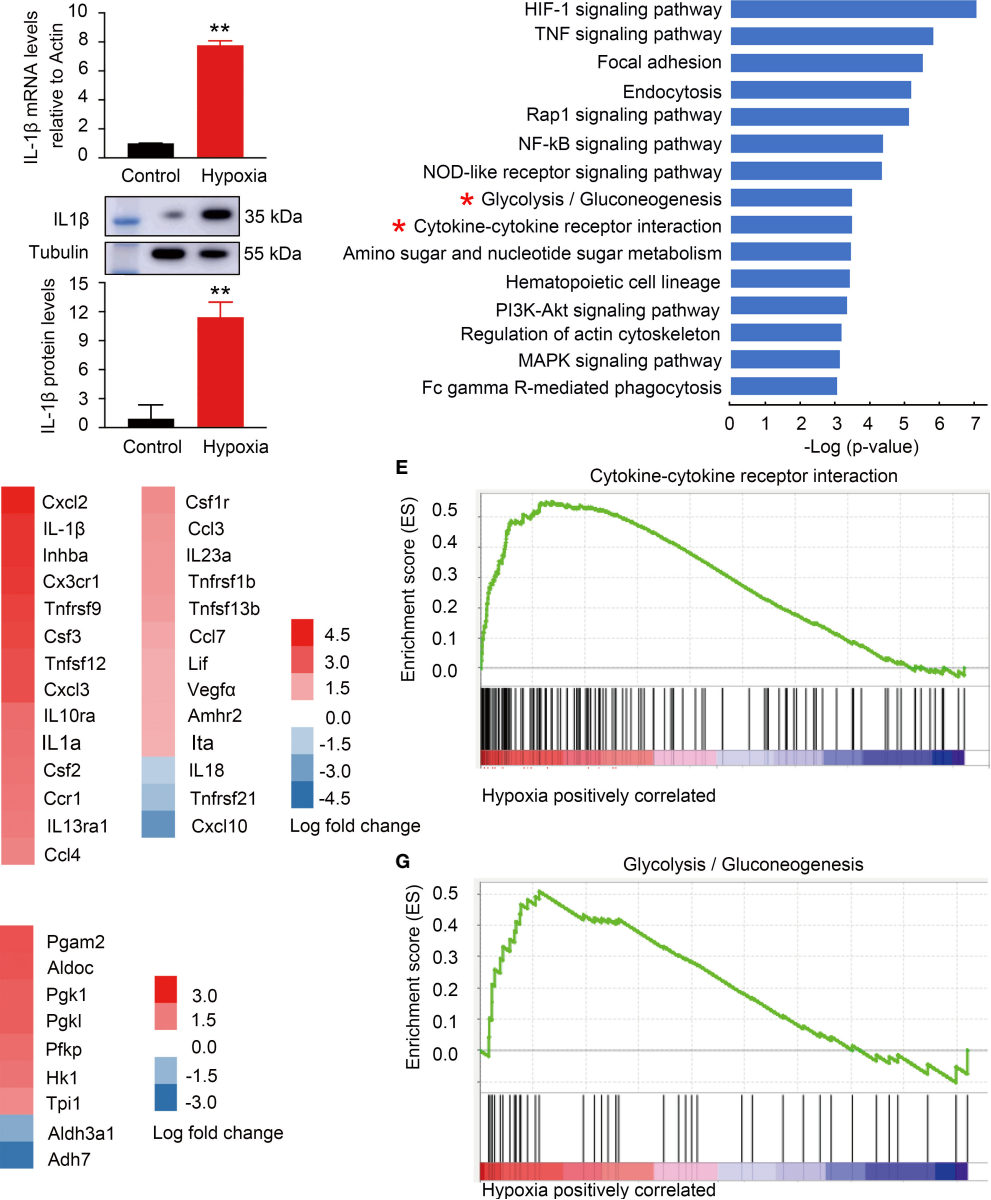
Hypoxia-induced BV2 activation with metabolic and inflammatory alterations mimicking activated retinal microglia (ARM) in DR. **(A,B)** Hypoxia-induced IL-1β expression at mRNA and protein levels using RT-PCR and western blot. **(C)** GSEA revealed inflammatory and metabolism-related pathways enriched in BV2 cells response to hypoxia. **(D,E)** Fold change heatmap of differential expressed genes in “Cytokine-cytokine receptor interaction” signature gene set against the ranked list of genes. Data represent the average of three control and three hypoxia stimulated cell samples. **(F,G)** Fold change heatmap of differential expressed genes enriched in “Glycolysis” signature gene set against the ranked list of genes. **p < 0.01.

To address the metabolic features of ARM with immune response, microglia cell line BV2 with hypoxia stimulation was established to mimic ARM in DR. Because the tissue hypoxia is associated with diabetes mellitus and responsible for DR development (11). Hypoxia-induced BV2 activation is manifested by increased cell proliferation, migration, phagocytosis, and increased proinflammatory factor production (1, 42). Here, the microglia activation was confirmed by the evidence of significant differential expression of IL-1β at mRNA and protein levels (**Figures 4A, B**). **Figure 4C** ranked top 20 canonical pathways with metabolic alterations especially glycolysis and inflammatory changes especially cytokine-related pathways enriched in the activated microglia. DEGs including IL-1β in the cytokine-related gene set were significantly upregulated along with glycolysis gene set (**Figures 4D–G**).

### Metabolomics profiles the intracellular metabolism alterations in the activated BV2 microglia

**Figure 5:**
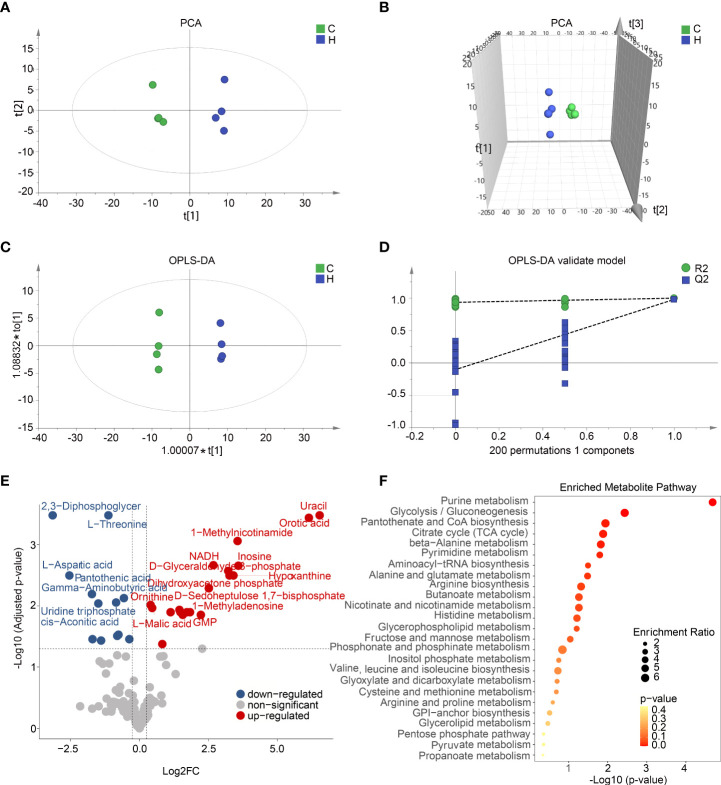
Activated microglia *in vitro* and *in vivo* with shared metabolic alterations including glycolysis and purine metabolism. **(A,B)** Two-dimensional **(A)** and three-dimensional **(B)** principal component analysis (PCA) score plots displayed a clear trend toward sample separation according activated BV2 cells and control. **(C)** Orthogonal partial least squares discriminant analysis (OPLS-DA) shows two clusters for activated BV2 cells and control groups. **(D)** Validation plot of OPLS model using a 200 times permutation test, and the R2-int and Q2-int values were 0.935 and −0.103, respectively. **(E)** The volcano plot showed the comparison of cell metabolites response to hypoxia induced microglia activation. The blue and red dots indicate metabolites downregulated and upregulated in hypoxia compared with control, respectively (fold change > 1.2, p < 0.05). **(F)** Pathway enrichment analysis of top 25 differential metabolites based on Kyoto Encyclopedia of Genes and Genomes (KEGG).

To profile the full extent of local metabolic shifts of activated microglia, metabolite profiling of hypoxia-induced immune response BV2 and control samples were analyzed by metabolomics. As indicated in **Figures 5A–D**, these two groups were separated in the score plot of PCA and orthogonal partial least squares discriminant analysis (OPLS-DA) models. A cluster of 200 permutated models was visualized using a validation plot, and the R2-int and Q2-int values were 0.935 and −0.103, respectively (**Figure 5D**). The validation plots from permutation tests strongly supported the validity of the established OPLS-DA model as all permuted R2 and Q2 values on the left were lower than the original point on the right, and the Q2 regression line in blue had a negative intercept. Seventy- eight variable metabolites were found to have variable important in projection (VIP) values higher than 1 and absolute correlation coefficients [i.e., p(corr)] higher than 0.5. By comparing the warped change values ​​for all metabolites, volcano plot analysis can easily filter out metabolites that vary significantly (**Figure 5E**). The metabolites with VIP value >1.0 and p-value <0.05 were identified as differential metabolites. Combining top intracellular altered metabolites and enriched metabolic pathways analysis, activated microglia strongly increased the levels of the purine metabolism intermediate hypoxanthine and inosine. All 40 differential metabolites were mainly significantly enriched in metabolic pathways such as purine metabolism and glycolysis (**Figure 5F**). The purine metabolic alterations of BV2 activation *in vitro* are similar to the metabolic features of STZ-induced ARM *in vivo* as shown in **Figure 3G**. Taken together, these results indicate a metabolic shift among BV2 microglia during activation.

### Lipidomics analysis reveals macroglia activation with local TAG accumulation

**Figure 6:**
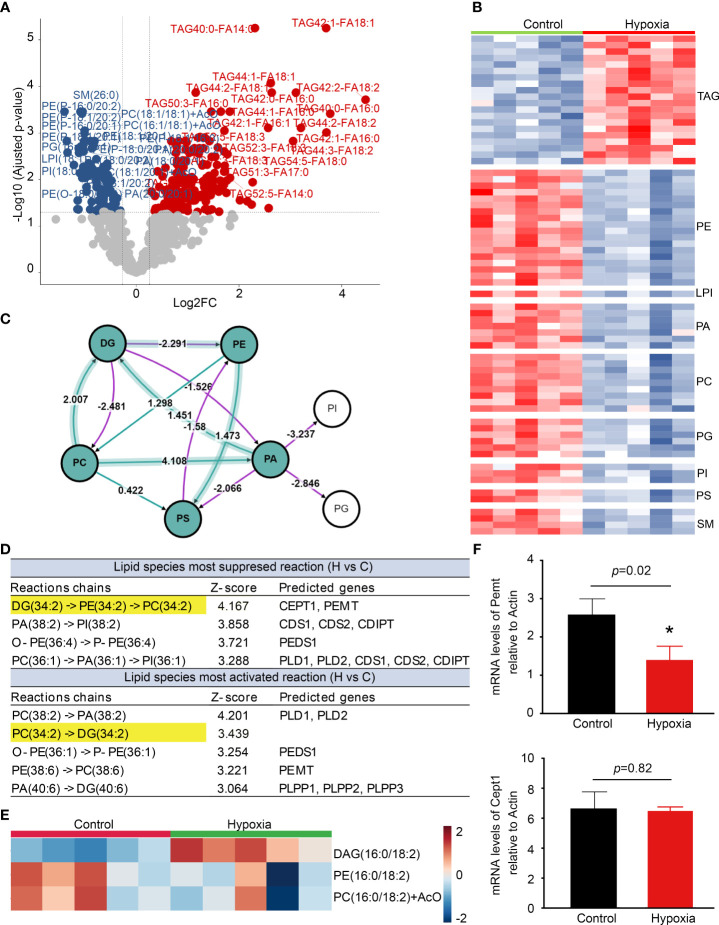
Lipidomics reveals microglia activation with triacylglycerol (TAG) accumulation. **(A)** The volcano plot shows the comparison of cell lipids between control and hypoxia. The blue and red dots indicate lipids downregulated and upregulated under hypoxia compared with control, respectively (fold change > 1.2, p < 0.05). **(B)** Heatmap representation of differentially abundance lipids. **(C)** Analysis of differential lipid pathway induced by hypoxia stress using BioPAN. **(D)** Lipid species most activated and suppressed in the hypoxia group compared with control. **(E)** Heatmap representation of diacylglycerol (DAG) -16:0/18:2, phosphatidylethanolamine (PE) -16:0/18:2, and phosphatidylcholines (PC) -16:0/18:2 +AcO (ant colony optimization) in the hypoxia and control groups. **(F)** Counts-per-million (CMP) values of Pemt and Cept1 expression in the control and hypoxia groups. *p < 0.05.

Lipidomics profiling indicated that the level of TAG was significantly increased, whereas phospholipids including phosphatidylethanolamine (PE), phosphatidic acid (PA), and phosphatidylcholine (PC) decreased markedly in the activated BV2 cells (**Figures 6A, B**). The lipidomics analysis was further performed by BioPAN to identify the activated or suppressed pathways associated with lipid metabolism and rank the reactions using Z-score values (**Figure 6C**). DG (34:2) metabolized to PC (34:2) was the most significantly suppressed reactions chains, and Pemt and Cept1 were predicted to play important roles in it (**Figure 6D**). Heatmap showed that DAG (34:2) levels were significantly increased in the activated BV2 cells (**Figure 6E**) with Pemt expression decrease compared with control (**Figure 6F**). These findings indicate that the activated microglia have significant lipid metabolism changes, especially TAG accumulation.

### Intracellular metabolic microenvironment may regulate IL-1β expression

**Figure 7:**
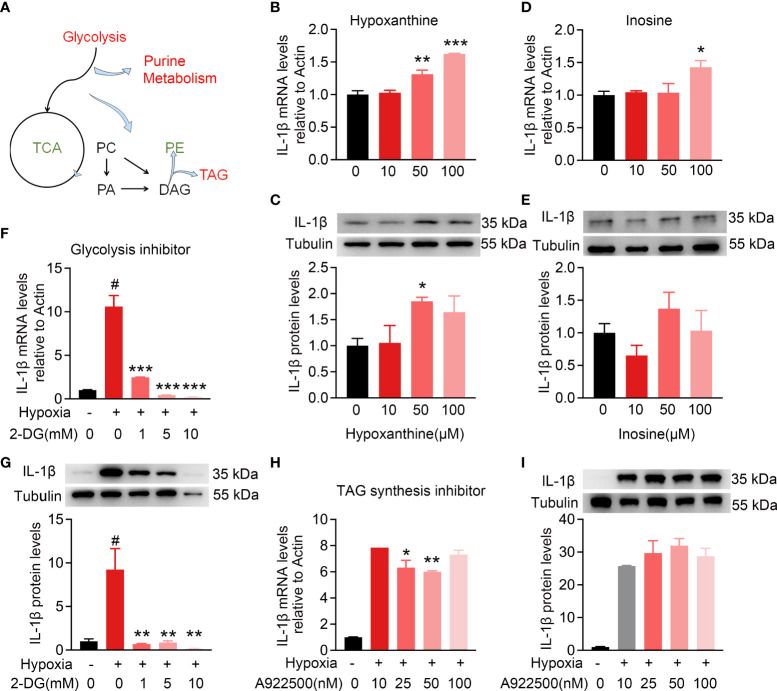
Intracellular metabolic microenvironment may regulate IL-1β expression of microglia. **(A)** Metabolic working model from metabolomics and lipidomics indicated hypoxia-induced microglia activation with intracellular glycolysis, purine metabolism, and TAG synthesis increase and TCA deceleration. **(B–E)** Purine metabolism intermediates hypoxanthine (10, 50, and 100 μM) and inosine (10, 50, and 100 μM) induced IL-1β expression in BV2 cells at different levels. **(F,G)** Hypoxia-induced IL-1β expression at RNA and protein level with or without glycolysis inhibitor 2-DG (1, 5, and 10 mM), respectively, using RT-PCR and Western blot. n = 3 for each group. *p < 0.05, **p < 0.01, and ***p < 0.001. **(H,I)** Hypoxia-induced IL-1β expression at RNA and protein levels with or without TAG synthesis inhibitor A922500 (10, 25, and 100 nM) using RT-PCR and Western blot. The meaning of # symbol is “no statistical difference”.

To further explore whether intracellular metabolic microenvironment contributes to pro-inflammation, the expression of IL-1β in BV2 cells was detected to assess the pro-inflammatory function among the top altered metabolites, including hypoxanthine and inosine, or inhibitors for metabolic pathways, including glycolysis and TAG synthesis isolated by metabolomics and lipidomics (**Figure 7A**). As shown in **Figures 7B, C** , hypoxanthine and inosine involved in purine metabolism were added to BV2 cells with indicated concentrations for 24 h. The mRNA expression levels of IL-1β had a dose-dependent increase in response to hypoxanthine treatment and only response to 100 μM inosine. In addition, the IL-1β protein level was significantly increased at 50 μM hypoxanthine and no significant change with the indicated treatment of inosine (**Figures 7D, E**). To probe the functional role of glycolysis in the activated microglia, 2-deoxyglucose (2-DG) was used as glycolysis inhibitor and robustly attenuated the hypoxia-inducible expression of IL-1β at mRNA and protein levels, compared with no inhibitor (**Figures 7F, G**); whereas treatment with the TAG synthesis inhibitor A922500 attenuated the hypoxia-inducible expression of IL-1β mRNA levels but no apparent changes at protein levels, compared with control (**Figures 7H, I**). These results indicate that the intracellular metabolic microenvironment especially glucose utilization may contribute to inflammation.

## Discussion

Here, we identified a subcluster of microglia with metabolism rewiring as the main origin of pro-inflammation factors especially IL-1β in the retina of STZ-induced DR mice. These activated microglia with IL-1β increase exhibited metabolic shifts including increased glycolysis, purine metabolism, and TAG synthesis but less TCA. In addition, some of these metabolites and involved metabolic pathways, especially glycolysis, were necessary to facilitate microglia pro-inflammation.

Inflammation is a critical driver of the pathophysiology of DR and plays an essential role at the early stage of DR (10, 43). Identifying the cellular contributors of inflammation in retina precisely could promote the development of potential treatment of early DR. However, it is controversial in previous *in vivo* histopathological studies and *in vitro* cell culture studies. Since the early morphology of macroglial and microglia change at the same time in diabetic rat’s retina (27), the rapid microglial activation and its typical morphological changes prior to macroglial activation in patients with diabetes (28) enhanced pro-inflammatory cytokines in vascular endothelial cells cultured with high glucose *in vitro* than Müller cells, astrocytes, and microglia (29). Therefore, we utilize scRNA-Seq to decode the heterogeneity in STZ-induced diabetic mice retina by generating transcriptomic profiles. Our results indicate that microglia are the main responder to increase the expression of pro-inflammatory especially IL-1β in the early DR (**Figure 2D**). This is similar to the conditions of DR human (28). Furthermore, scRNA-Seq analysis enriched a subcluster of activated microglia named cluster 36 with an increased number and majority of IL-1β and Tnf expression in STZ-induced diabetic retina (**Figure 2F**). It matched the properties of microglia activation in the retina that involve proliferation and inflammation (44). The increasing numbers of moderately hypertrophic microglial cells in STZ-induced mice retina as shown in **Figures 3A, B** are similar to the patients with non-proliferative diabetic retinopathy (NPDR) by histological studies (45). Moreover, IL-1β, Tnf, and IL6 were expressed in the whole retina of patients with DR as well (12–15). On the basis of our scRNA-Seq analysis, microglia were also the major sources of Tnf in STZ-induced DR mice retina, although their abundance was lower than IL-1β (**Figure 2D**). Whereas, the expression of IL6 was elevated in STZ astrocytes according to our scRNA-Seq analysis (data not shown). More experiments are needed to define the pathological functions of IL6 induction in diabetic retinal astrocytes in the future. Hence, our study identifies a subcluster of activated microglia as the main cell targets to produce pro-inflammatory cytokines in the early DR. Microglia are a key cell population that triggers retinal inflammation in autoimmune uveitis, and depletion of microglia completely blocks the neuroinflammation initiated by retinal microglia in ocular autoimmunity (46). Therefore, removing these activated microglia or inhibiting their activation might develop potential approaches for the prevention and treatment of early DR.

An increasing number of studies demonstrated that microglia undergo a chain of metabolic events during microglia activation (47). Metabolism and immune system are the most fundamental requirements for biology. In this way, immune response and metabolic regulation are highly integrated and interdependent each other (48). Here, we investigate whether the intracellular metabolic microenvironment contributes to immune mechanisms, because the ARM enriched in cluster 36 show major disturbances of metabolic and inflammatory changes, and it is similar to the hypoxia-induced activated microglia (**Figures 3**–**6**). With integrated metabolomics, lipidomics and functional test of proinflammatory cytokines expression in microglia cell line samples treating with metabolites or inhibitors for key enzymes of the metabolic pathways, our results show that activated microglia with IL-1β increase exhibited a metabolic bias favoring glycolysis, purine metabolism, and TAG synthesis but less TCA (**Figures 4**, **5**). In addition, glycolysis with 2-DG inhibition of IL-1β expression induced by hypoxia was necessary to facilitate their pro-inflammation in activated microglia (**Figures 7F, G**). Similarly, 2-DG inhibits the activation of macrophages in the lipopolysaccharides (LPS)-induced acute lung injury model in mice by reducing the inflammatory factors (49). 2-DG administration attenuates IgG-related renal macrophage activation, pro-inflammatory cytokine secretion, and renal inflammation in nephritis mice (50). Moreover, 2-DG is currently proposed to inhibit immune cell glycolysis and calm the cytokine storm in COVID-19 (51). Further *in vivo* studies are required to determine whether 2-DG could attenuate inflammation in the early stage of DR mice. Moreover, phosphoglycerate mutase (PGAM), as shown in differential expressed genes of glycolysis/gluconeogenesis in **Figure 4F**, catalyzed the conversion of triphosphoglycerate to diphosphoglycerate. However, its role in DR remains unclear. Interestingly, overexpression of Pgam in mice hearts induces an inflammatory response and impairs cardiac function (52). Whereas, PGAM inhibitors decrease the proliferation and migration of tumor tissues in breast cancer and lung cancer (53). Similar to PGAM, targeting phosphoglycerate kinase 1 (PGK1) to inhibit glycolysis has become a new therapeutic target in tumor tissues through regulating inflammation (50, 54), and inhibition of PGK1 in macrophages can also reduce the production of inflammatory factors in lung cancer (55). As described above, further exploring the underlying mechanisms and potential targets of glycolysis in the early DR may offer promising approaches for DR treatment.

Purine metabolism ranks the top changed pathways in ARM in STZ-induced DR mice and microglia cell lines as shown in scRNA-Seq and metabolomic data (**Figures 3**, **5**). In addition, metabolomics analysis showed that hypoxanthine and inosine, the metabolites involved in purine metabolism, are significantly increased in activated microglia *in vitro* (**Figure 4**). They are consistent with the enhanced levels of inosine and other purine-related metabolites in the serum of type 2 diabetic subjects with the progression of DR and in the vitreous of patients with DR (56, 57). Moreover, with the correlations above, our data further evaluated the role of intracellular purine metabolites in the inflammation and indicated that the elevated hypoxanthine promotes the expression of IL-1β in microglia in a dose-dependent manner (**Figures 7C, D**). Consistent with our results, intrastriatal administration of hypoxanthine, a metabolite accumulated in Lesch-Nyhan syndrome, increased neuroinflammatory parameters in the striatum of rats by activating the NF-kB pathway (58). Furthermore, hypoxanthine had been reported as a hypoxic metabolite and a checkpoint stress metabolite for cellular energy regulation (59). Therefore, the increase of intracellular hypoxanthine might indicate that ARM is in a state of hypoxia.

Activated macrophages undergo comprehensive remodeling of lipid metabolism. In our study, activated microglia accumulates large amounts of triglycerides (**Figure 6A**). Similarly, in the LPS-activated macrophages, the synthesis of TAG was significantly increased (60). When treated with the inhibitors of DGAT1, a key enzyme in TAG synthesis, the inflammatory factors including IL-1β, IL-6, and PGE2 are reduced markedly. Therefore, the accumulation of TAG in activated microglia may serve as a target for the inflammation in DR. As shown in **Figure 6**, the expression of PE N-methyltransferase (PEMT), an enzyme to catalyze the methylation of PE to PC in cells, is significantly decreased in microglia activated accompanied with a decreased level of PC (**Figure 6**). Whereas, the functions of PEMT in early DR remain unknown. Studies show that PEMT alters the hepatic ratio of PC/PE and affects inflammation through endoplasmic reticulum stress (61). In addition, Pemt knockout mice prevent diabetic nephropathy by ameliorating endoplasmic reticulum stress and subsequent inflammation (62). These suggest a possible function of PEMT in DR. Type 1 diabetic mellitus (T1DM) and T2DM could both lead to DR. It is reported that lipid lowering therapy with statins protected against the progression of DR in T2DM (63). Moreover, the use of inhibitors of lovastatin 3-hydroxy-3-methylglutaryl-coenzyme A, a rate-limiting step enzyme in cholesterol biosynthesis, reduced the expression of inflammatory factors Tnf and Icam-1 in T2DM mouse model (64).

In conclusion, we identified the activated microglia with metabolic features in retina that were the main source of retinal pro-inflammatory cytokines in the early DR. Intracellular metabolic microenvironment especially glycolysis might reprogram the retinal inflammation. For future studies, it was proposed to be conceivable to target intracellular metabolic reprogramming of retinal specific microglia to reduce local inflammation and thereby prevent the early DR progression.

## Data availability statement

The datasets presented in this study can be found in online repositories. The names of the repository/repositories and accession number(s) can be found below: https://www.ncbi.nlm.nih.gov/geo/, GSM5380581; https://www.ncbi.nlm.nih.gov/geo/, GSM5380582.

## Ethics statement

The animal study was reviewed and approved by Animal Care and Use Committee of Shanghai General Hospital.

## Author contributions

The authors confirm contribution to the paper as follows: Study conception and design: FZ, KL, and HY; data collection: KL, GH, and QJ; analysis and interpretation of results: KL, HY, and JH; draft manuscript preparation: KL and HY. All authors reviewed the results and approved the final version of the manuscript.

## Funding

This work was supported by Training Program of the Major Research Plan of the National Natural Science Foundation of China (92057106), Natural Science Foundation of China (32171177), Natural Science Foundation of Shanghai (19ZR1440500), and Shanghai Jiaotong University-Gaofeng Clinical Medicine Grant Support, Shanghai Pujiang Program (2019PJD046).

## Conflict of interest

The authors declare that the research was conducted in the absence of any commercial or financial relationships that could be construed as a potential conflict of interest.

## Publisher’s note

All claims expressed in this article are solely those of the authors and do not necessarily represent those of their affiliated organizations, or those of the publisher, the editors and the reviewers. Any product that may be evaluated in this article, or claim that may be made by its manufacturer, is not guaranteed or endorsed by the publisher.
